# Amelioration of Rheumatoid Arthritis by *Anacardium occidentale* via Inhibition of Collagenase and Lysosomal Enzymes

**DOI:** 10.1155/2020/8869484

**Published:** 2020-11-07

**Authors:** Rabiya Naz, Zaheer Ahmed, Muhammad Shahzad, Arham Shabbir, Faiza Kamal

**Affiliations:** ^1^Department of Home and Health Sciences, Allama Iqbal Open University Islamabad, Islamabad, Pakistan; ^2^Department of Pharmacology, University of Health Sciences Lahore, Lahore, Pakistan; ^3^Institute of Pharmacy, Faculty of Pharmaceutical and Allied Health Sciences, Lahore College for Women University, Jail Road, Lahore, Pakistan

## Abstract

*Anacardium occidentale* (cashew) has been used in the traditional system of medicine for curing many inflammatory disorders. The present study investigates the antiarthritic effects of cashew leaves extract using the rat model of FCA-induced rheumatoid arthritis. Arthritic rats were treated with 100 and 200 mg/kg b.w. ethanolic extract of cashew leaves. Animals were sacrificed at day 23, and before sacrificing the animals, gross pathological changes were observed. Histopathology of ankle joint was evaluated with hematoxylin and eosin staining, whereas the serum levels of C-reactive protein (CRP) were evaluated by the agglutination method. Inflammatory cells and other hematological parameters were assessed by employing an automated hemocytometer and chemistry analyzer. Rheumatoid factor (Rf) and lysosomal enzymes levels were determined in blood. Results indicated that *A. occidentale* significantly decreased the CPR levels, macroscopic arthritic score, and rheumatoid factor as compared to the diseased group. Histopathological evaluation showed significant attenuation in bone erosion, joint inflammation, and pannus formation by plant extract. Treatment with *A. occidentale* significantly suppressed the levels of acid phosphatase, *β*-galactosidase, *β*-glucuronidase, *N*-acetylglucosaminidase, and collagenase. Moreover, *A. occidentale* significantly raised the HB levels and RBCs counts which were found depleted in the diseased group. The raised counts of total leukocytes, platelets, neutrophils, lymphocytes, and monocytes were also significantly decreased by treatment with plant extract. Comparative analysis showed that higher dose of *A. occidentale* demonstrated superior amelioration of rheumatoid arthritis as compared to low dose. In conclusion, *A. occidentale* possesses significant antiarthritic potential, which may be attributed to the suppression of lysosomal enzymes and collagenase levels.

## 1. Introduction

Rheumatoid arthritis is an autoimmune disease that affects the joints but can also involve various extra-articular complications. It affects almost every diarthrodial joint and the hallmark features include persistent chronic inflammation, pannus formation, cartilage/bone erosion, and synovial proliferation. Extra-articular manifestations may implicate weight loss, rheumatoid nodules, vasculitis, serositis, etc. [1–3]. Globally, 1% of the population suffers from the rheumatoid arthritis and the age between 35 and 45 years is considered as the peak age for the onset of disease. RA significantly affects the quality of life causing elevation in functional and work disability, morbidity rate, and economic burden [4–6].

Conventional medicines currently in use for arthritic treatment, such as disease-modifying antirheumatic agents, NSAIDs, steroids, and biological agents, have only limited success against RA and are linked with numerous side effects [7, 8]. Long-term use of these therapies is associated with well-known limitations like risks of hematological, gastrointestinal, cardiovascular, and kidney disorders and loss of response [9]. Plant-based medicines are gaining prime importance among patients suffering from RA due to the adverse effects associated with conventional therapy [10, 11].

*Anacardium occidentale* L. (Family: Anacardiaceae) has been traditionally used for curing many inflammatory diseases [12]. Previous studies have shown anti-inflammatory effects of *A. occidentale* in different models of inflammation. It is known to inhibit carrageenan-induced paw edema and formalin-induced paw licking in rats [13]. Treatment with *A. occidentale* also reduced ear edema and impaired leukocyte migration into the peritoneal cavity when tested using carrageenan-induced peritonitis [14]. Methanolic extract of *A. occidentale* showed protection against lipopolysaccharide-induced microvascular permeability [15, 16]. Treatment with *A. occidentale* significantly reduced prostaglandin-E2 production in the microglia. The plant showed anti-inflammatory property by inhibiting inflammation-associated cytokine production. Moreover, significant inhibition of COX-2 and iNOS gene production by blocking MAPK and NF-*κ*B pathways were observed [16]. The current study aimed to evaluate the antiarthritic effects of *Anacardium occidentale* using FCA-induced rat model of arthritis.

## 2. Materials and Methods

### 2.1. Experimental Animals

Male Wistar rats of age 6 to 8 weeks, weighing 225–250 g, were retained at standard conditions in animal house of the University of Punjab, Lahore. Animals were given distilled water and standard rat chow *ad libitum*. After development of arthritis, food was given on the lower portion of the cages as severely arthritic rats have problem in taking feed from the upper half of the cage. Standard temperature and humidity conditions (24–26°C and 40–60%, resp.) were maintained [17]. All the experiments and protocols were approved by the Allama Iqbal Open University, Islamabad (AIOU/283).

### 2.2. Preparation of Plant Extract

The leaves of the *Anacardium occidentale* were collected, identified by the Botanist, and shade-dried at room temperature. The leaves were ground to powdered form (yielding 300 g) and the powdered sample was macerated in 2 L of ethanol at room temperature for 24 h. Filtrate was collected by passing the mixture through muslin cloth and subsequently through filter paper (Whatman No. 1). The residue left was again macerated in ethanol and the procedure was repeated 3 times for the collection of filtrates. The resultant filtrate was evaporated in water bath (maintained at 40°C) to obtain a semisolid extract. The percentage yield was calculated as 6.67%. The extract was then kept in a refrigerator at 4°C for further use.

### 2.3. Experimental Design and Induction of Arthritis

Rats were distributed into 5 groups, having 6 rats in each group.

  Group 1 (control): it included healthy rats serving as control group, injected with 0.1 ml normal saline at the subplantar region in hind paw.
  Group 2 (arthritic): Freund's Complete Adjuvant (FCA) was used to induce arthritis. Normal saline was administered to this group from day 8, once daily for 15 days.
  Group 3 (AO 100 mg/kg): it included arthritic rats receiving low oral dose of *Anacardium occidentale* extract, i.e., 100 mg/kg b.w. Treatment was started from day 8, once daily for 15 days.
  Group 4 (AO 200 mg/kg): arthritic rats were given high oral dose of *Anacardium occidentale* extract, i.e., 200 mg/kg b.w. Treatment was started from day 8, once daily for 15 days.
  Group 5 (piroxicam): arthritic rats received 10 mg/kg b.w. piroxicam as standard drug [18]. Treatment was started from day 8, once daily intraperitoneally for 15 days.

For the induction of arthritis, 0.2 ml FCA (a suspension of heat-killed *Mycobacterium*) was injected in the subplantar region of the left hind rat paw on day 0 in all groups except control group. Treatment was started at the 8th day of arthritis induction for 15 days and all animals were sacrificed at day 23 [18].

### 2.4. Determination of Arthritic Development

Incidence and severity of arthritis was evaluated by arthritic scoring method. Presence of the detectable clinical arthritic features characterized by edema and/or erythema in the hind rat paws was observed. Through macroscopic evaluation, arthritic score was determined at days 13, 18, and 23. Scores 0–4 were given to normal-severe edema and/or erythema in ipsilateral paw. Arthritic score crossing 4 is the indication of involvement of contralateral paw [19].

### 2.5. Histopathological Examinations of Ankle Joints

The ankle joints of the ipsilateral paw were cut and fixed in 10% formalin for 36 hours. These separated joints were then submerged in decalcifying solution (a mixture of ethylenediamine tetra acetic acid, sodium tartrate, hydrochloric acid, and potassium sodium tartrate) for 48 hours. Collected tissues were further treated for implanting in paraffin blocks, sliced at 5 *μ*m thickness, and stained with hematoxylin and eosin (H&E). The histopathologist studied the slides in blinded fashion for the detection of bone erosion, inflammation, and pannus formation. A scale (0 to 4) was used for recording results, where 0 represents no pathological change whereas scores 1 to 4 refer to minimal, mild, moderate, and severe changes, respectively [17, 20].

### 2.6. Determination of Inflammatory Cell and Other Hematological Parameters in Blood

At the time of dissection, blood was collected in EDTA containing tubes by intracardiac puncture. Inflammatory cell counts like eosinophils, neutrophils, basophils, and lymphocytes were evaluated in Giemsa Wright stained blood smears under light microscope [21], and counts of RBCs, platelets, and total leukocytes along with Hb content were determined by using automated hemocytometer.

### 2.7. Evaluation of C-Reactive Protein Levels

On day 23, all the rats were sacrificed and blood was withdrawn by intracardiac puncture. Serum was collected after centrifugation for 10 min at 2380 g. The levels of CPR were determined by agglutination method using commercially available kits (Antec Diagnostic Products, UK). Significant agglutination is observed when high amount of CPR is preset in the serum and it interacts with the antisera. CRP levels were semiquantified according to the kit's protocol [3].

### 2.8. Determination of Rheumatoid Factor Levels

Rheumatoid factor is the autoantibody produced in arthritis. The level of rheumatoid factor (Rf) was estimated by means of the latex method. The complete procedure was followed according to the manufacturer's guidelines (Approach Bioscience). The concentration of rheumatoid factor was expressed as IU/ml [22].

### 2.9. Determination of Lysosomal Enzymes and Collagenase Levels

Acid phosphatase analysis was performed by utilizing a substrate known as disodium phenyl phosphate [23]. *β*-Glucuronidase was determined by the method of Kawai and Anno [24]. *p*-Nitrophenyl *β*-D-glucuronide was used as enzyme substrate in the reaction. *N*-Acetylglucosaminidase was measured by using the substrate 4-nitrophenyl *N-*acetylglucosaminide [25]. The activity of *β*-galactosidase was assessed by the method of [26] by utilizing the substrate 4-nitrophenyl *N*-acetyl galactopyranoside. Protein concentration was determined as described by [27]. Activity of collagenase was measured by the method of Van and Steinbrink [28], using *N*-(3-[2-furyl] acryloyl)-Leu-Gly-Pro-Ala as substrate.

### 2.10. Statistical Analysis

The data was analyzed using GraphPad version 5 software. Mean ± standard deviation was used to represent the data. One-way analysis of variance and post hoc Tukey's test was applied to compare the quantitative variables and to analyze the difference among all groups. *P* < 0.05 was considered as statistically significant.

## 3. Results

### 3.1. *A. occidentale* Suppressed the Arthritic Development

**Figure 1:**
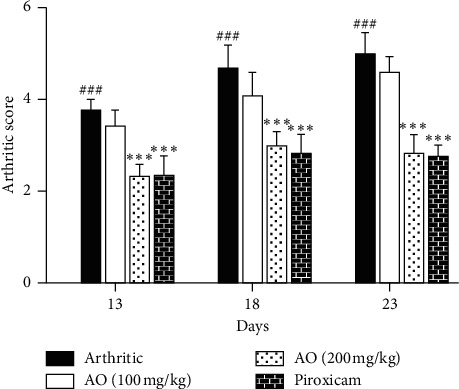
Treatment with *A. occidentale* showed significant reduction in arthritic score. The data is presented as mean ± SD for *n* = 6. ^###^(*p* < 0.001) indicating significant difference compared to control group, while ^*∗∗∗*^(*p* < 0.001) indicates significant difference compared to arthritic group.

The results indicated a significantly high arthritic score in arthritic rats as compared to control on day 13 (0.00 ± 0.00 vs. 3.750 ± 0.2739), day 18 (0.00 ± 0.00 vs. 4.667 ± 0.5164), and day 23 (0.00 ± 0.00 vs. 5.000 ± 0.4472). Although treatment with low dose of *A. occidentale* reduced the arthritic score on all these days, this reduction was not statistically significant as compared to the arthritic group. High dose of *A. occidentale* significantly reduced (*p* < 0.001) the arthritic score on day 13 (2.333 ± 0.2582 vs. 3.750 ± 0.2739), day 18 (3.00 ± 0.3162 vs. 4.667 ± 0.5164), and day 23 (2.833 ± 0.4082 vs. 5.00 ± 0.4472). Piroxicam also significantly decreased (*p* < 0.001) the arthritic score on these days as compared to the arthritic group (2.333 ± 0.4082 vs. 3.750 ± 0.2739), (2.833 ± 0.4082 vs. 4.667 ± 0.5164), and (2.750 ± 0.2739 vs. 5.000 ± 0.4472), respectively. There was no significant difference in the determined arthritic scores in high-dose plant extract-treated group and piroxicam-treated group on all days. The arthritic scores determined at days 13, 18, and 23 are presented Figure 1.

### 3.2. *A. occidentale* Significantly Suppressed C-Reactive Protein Levels

**Figure 2:**
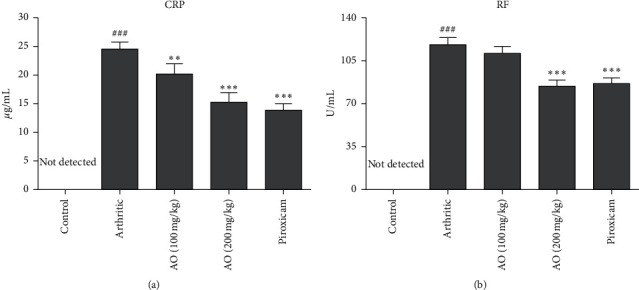
Treatment with *A. occidentale* extract caused significant suppression of levels of CRP and RF. The data is presented as mean ± SD for *n* = 6. ^###^(*p* < 0.001) indicating significant difference compared to control group, while ^*∗∗*^(*P* < 0.01) and ^*∗∗∗*^(*p* < 0.001) indicate significant difference compared to arthritic group.

Administration of FCA resulted in increased CRP levels of arthritic group as compared to control group (24.48 ± 1.362 vs. 0.00 ± 0.00). Both low- and high-dose treatment of *A. occidentale* showed a significant reduction of CRP levels as compared to arthritic group (20.08 ± 1.931 vs. 24.48 ± 1.362) and (15.26 ± 1.727 vs. 24.48 ± 1.362), respectively. Similarly, piroxicam treatment also significantly decreased the CRP levels as compared to arthritic group (13.76 ± 1.172 vs. 24.48 ± 1.362). On comparing three treatment groups, we found that piroxicam showed a significantly higher capacity to alleviate C-reactive protein levels as compared to the low dose of *A. occidentale*, while no significant difference was seen when compared with the high dose of *A. occidentale* (Figure 2(a)).

### 3.3. *A. occidentale* Significantly Inhibited Rheumatoid Factor (RF)

Increased levels of RF were found in arthritic group as compared to control group (119.7 ± 5.955 vs. 0.00 ± 0.00). Both high dose of *A. occidentale* and piroxicam showed a significant reduction in RF levels as compared to arthritic group (86.00 ± 4.099 vs. 119.7 ± 5.955) and (87.67 ± 4.546 vs. 119.7 ± 5.955), respectively. Low dose of *A. occidentale* did not show significant reduction in RF levels compared to arthritic group (112.0 ± 5.762 vs. 119.7 ± 5.955). We did not find any significant difference between the effect of high-dose *A. occidentale* and piroxicam (86.00 ± 4.099 vs. 119.7 ± 5.955) (Figure 2(b)).

### 3.4. Effects of *A. occidentale* on Histopathological Changes in Ankle Joints

**Figure 3:**
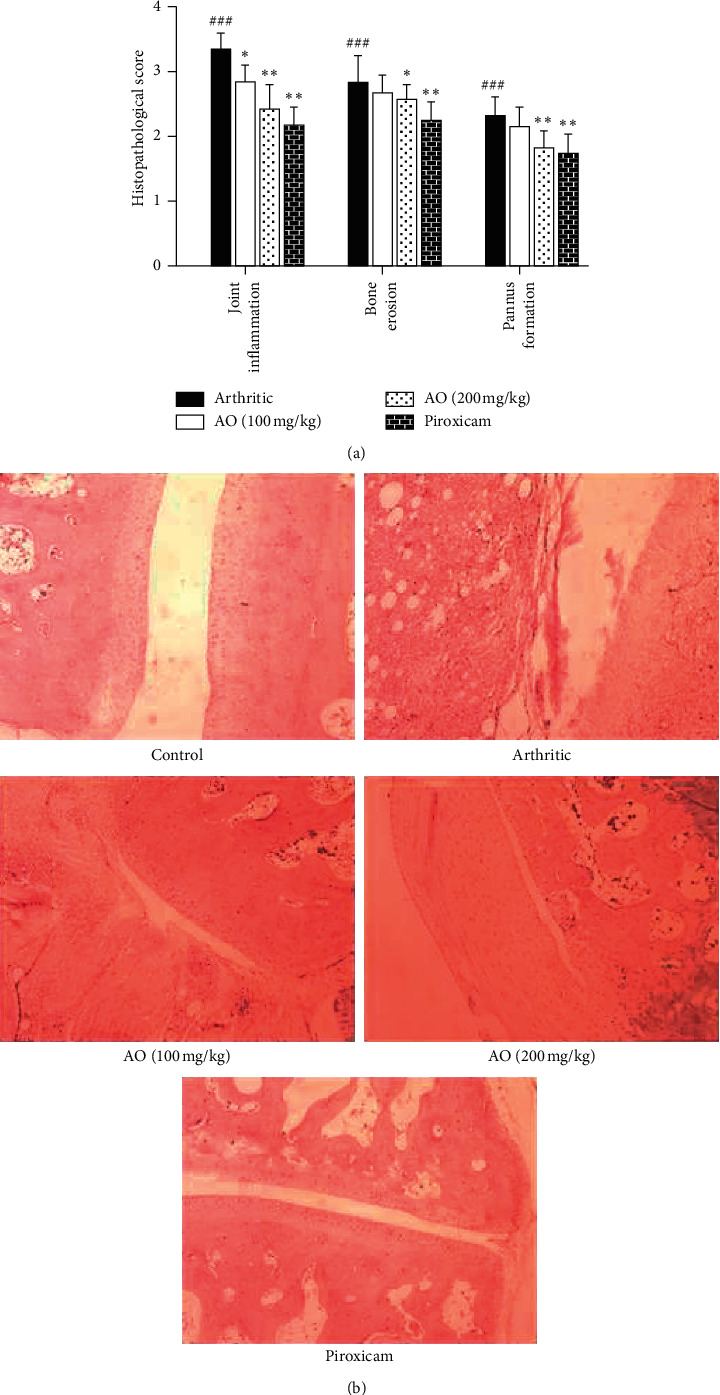
(a) Treatment with *A. occidentale* significantly reduced the histopathological score. The data is presented as mean ± SD for *n* = 6. ^###^(*p* < 0.001) indicating significant difference compared to control group, while ^*∗*^(*P* < 0.05), ^*∗∗*^(*P* < 0.01), and ^*∗∗∗*^(*p* < 0.001) indicate significant difference compared to arthritic group. (b) H&E staining, showing normal ankle joint tissue, normal synovium, cartilage, bone, and no inflammation (control group); severe inflammation, pannus, and bony erosion (arthritic group); resolution of inflammation, pannus formation, and bony erosion (low-dose and high-dose AO group); and resolution of inflammation, pannus formation, and bony erosion (piroxicam group).

Severe inflammation along with massive joint inflammation, bone erosion, and pannus formation was observed in paw tissue of arthritic rats. Treatment with *A. occidentale* and piroxicam significantly decreased these histopathological changes (Figures 3(a) and 3(b)).

#### 3.4.1. *A. occidentale* Significantly Attenuated Joint Inflammation

Significant joint inflammation was observed in arthritic group as compared with control group (3.333 ± 0.2582 vs. 0.0 ± 0.0). Treatment with low dose and high dose of *A. occidentale* (2.833 ± 0.2582 vs. 3.333 ± 0.2582) and (2.417 ± 0.3764 vs. 3.333 ± 0.2582) and piroxicam (2.167 ± 0.2582) significantly reduced inflammation.

#### 3.4.2. Treatment with *A. occidentale* Significantly Alleviated Bone Erosion

We observed significantly high bone erosion in arthritic group as compared to control group (2.833 ± 0.4082 vs. 0.0 ± 0.0). Although treatment with low dose of *A. occidentale* decreased the bone erosion, it was not significant (2.667 ± 0.2582 vs. 2.833 ± 0.4082). High-dose *A. occidentale* and piroxicam significantly reduced the bone erosion (2.583 ± 0.2041 vs. 2.833 ± 0.4082) and (2.250 ± 0.2739 vs. 2.833 ± 0.4082), respectively.

#### 3.4.3. *A. occidentale* Significantly Suppressed Pannus Formation

Results showed increased pannus formation in arthritic group (2.333 ± 0.2582) as compared to control group. Treatment with low dose of *A. occidentale* did not significantly decrease the pannus formation (2.167 ± 0.2582 vs. 2.333 ± 0.2582) while high dose of *A. occidentale* and piroxicam significantly reduced the pannus formation as compared to arthritic group (1.833 ± 0.2582 vs. 2.333 ± 0.2582) and (1.750 ± 0.2739 vs. 2.333 ± 0.2582), respectively.

### 3.5. *A. occidentale* Nearly Normalized the Total Leucocyte Count (TLC)

The results showed an increase in TLC of arthritic group as compared to control group (9.293 ± 0.569 vs. 7.305 ± 0.3885) which means FCA-induced arthritis resulted in recruitment of leukocytes in arthritic group. Both low-dose and high-dose treatment of *A. occidentale* showed a significant decrease in TLC as compared to arthritic group (8.017 ± 0.3214 vs. 9.293 ± 0.569) and (7.900 ± 0.4801 vs. 9.293 ± 0.569), respectively. Similarly, piroxicam-treated group also showed a significant decrease in TLC as compared to arthritic group (7.807 ± 0.6639 vs. 9.293 ± 0.569). There was no significant difference in the TLC of low dose, high dose, and piroxicam groups when compared to each other (Table 1).

### 3.6. *A. occidentale* Significantly Suppressed the Raised Levels of DLC

#### 3.6.1. *A. occidentale* Significantly Reduced Neutrophil Count

There was a significant increase in neutrophil count of arthritic group as compared to control group (66.78 ± 2.660 vs. 52.66 ± 3.369). Both low-dose and high-dose treatment of *A. occidentale* showed a significant decrease in neutrophil count as compared to arthritic group (60.43 ± 3.972 vs. 66.78 ± 2.660) and (57.94 ± 3.052 vs. 66.78 ± 2.660), respectively. Similarly, piroxicam-treated group also showed a significant decrease in TLC as compared to arthritic group (54.50 ± 3.355 vs. 66.78 ± 2.660). On comparing three treatment groups, we found that piroxicam showed a significantly higher capacity to alleviate neutrophil count as compared to low dose of *A. occidentale* while no significant difference was observed when compared with high dose of *A. occidentale* (Table 1).

#### 3.6.2. *A*. *occidentale* Significantly Reduced the Elevated Lymphocytes Counts

We found a significant increase in lymphocyte counts of arthritic group as compared to control group (33.66 ± 0.7083 vs. 31.18 ± 0.6081) which indicates that FCA-induced arthritis resulted in recruitment of lymphocytes in arthritic group. Low dose of *A. occidentale* could not significantly reduce the lymphocyte count (33.27 ± 1.693 vs. 33.66 ± 0.7083), while high-dose treatment of *A. occidentale*, as well as piroxicam, showed a significant decrease in lymphocyte count as compared to arthritic group (32.02 ± 0.5516 vs. 33.66 ± 0.7083) and (31.96 ± 0.5322 vs. 33.66 ± 0.7083), respectively. No significant difference was noted between the effects of high-dose *A. occidentale* and piroxicam when compared with one another (Table 1).

#### 3.6.3. *A. occidentale* Ameliorated the Altered Monocyte Counts

A significant increase in monocyte counts of arthritic group was determined as compared to control group (2.412 ± 0.2001 vs. 1.610 ± 0.1336). Low dose of *A. occidentale* did not significantly reduce the lymphocyte count (2.303 ± 0.1720 vs. 2.412 ± 0.2001), while high-dose treatment of *A. occidentale*, as well as piroxicam, showed a significant decrease in monocyte count as compared to arthritic group (2.088 ± 0.1179 vs. 2.412 ± 0.2001) and (2.032 ± 0.1466 vs. 2.412 ± 0.2001), respectively. No significant difference was found between the effect of high dose *A. occidentale* and piroxicam when compared with one another (Table 1).

#### 3.6.4. No Significant Change Was Observed in the Counts of Basophils

Results showed no significant change in basophil counts of arthritic group, low-dose *A. occidentale*, high-dose *A. occidentale*, and piroxicam-treated groups, when compared with each other (Table 1).

### 3.7. *A. occidentale* Reduced the Raised Platelet Counts

A significant increase in platelet count of arthritic group was observed as compared to control group (5.567 ± 0.3259 vs. 4.350 ± 0.2504). Low dose of *A. occidentale* did not significantly affect the platelet count (5.133 ± 0.1894 vs. 5.567 ± 0.3259), while high-dose treatment of *A. occidentale* (4.908 ± 0.3642 vs. 5.567 ± 0.3259), as well as piroxicam (4.882 ± 0.1847 vs. 5.567 ± 0.3259), showed a significant decrease in platelet count as compared to arthritic group. No significant difference was found between the effects of high-dose *A. occidentale* and piroxicam when compared with each other (Table 1).

### 3.8. *A. occidentale* Significantly Improved the RBC Count

Results indicated a significant decrease in RBC count of arthritic group as compared to control group (4.163 ± 0.4052 vs. 5.827 ± 0.3257). Low dose of *A. occidentale* did not significantly restore the RBC count (4.450 ± 0.4241 vs. 4.163 ± 0.4052), while high-dose treatment of *A. occidentale* and piroxicam showed a significant increase in RBC count as compared to arthritic group (5.057 ± 0.3642 vs. 4.163 ± 0.4052) and (4.963 ± 0.1445 vs. 4.163 ± 0.4052), respectively (Table 1).

### 3.9. *A. occidentale* Restored the Hemoglobin Content

There was a significant decrease in Hb content of arthritic group as compared to control group (11.67 ± 0.8179 vs. 14.66 ± 0.3985). Low dose and high dose of *A. occidentale* significantly restored the Hb content as compared to arthritic group (12.79 ± 0.3164 vs. 14.66 ± 0.3985) and (13.36 ± 0.4298 vs. 14.66 ± 0.3985), respectively. Similarly, piroxicam also significantly improved the Hb content (13.02 ± 0.6174 vs. 14.66 ± 0.398). There was no significant difference in the effects of high dose of *A. occidentale* and piroxicam (Table 1).

### 3.10. Effects of *Anacardium occidentale* on Lysosomal Enzymes

#### 3.10.1. Treatment with *A. occidentale* Significantly Reduced the Acid Phosphatase Levels

**Figure 4:**
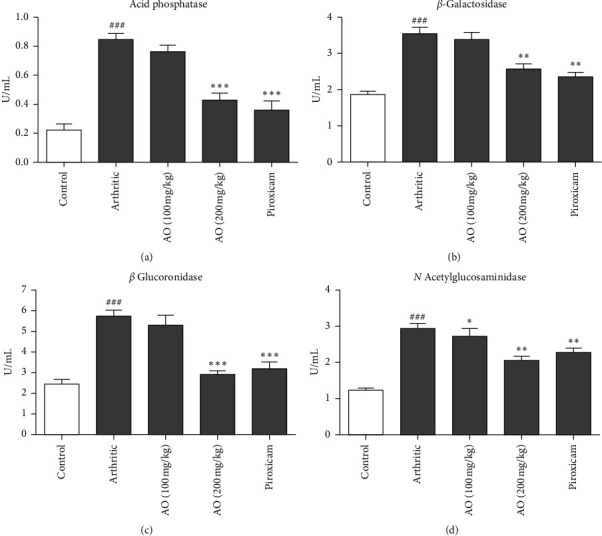
Treatment with *A. occidentale* leaves extract significantly reduced acid phosphatase, *β*-galactosidase, *β*-glucuronidase, and *N*-acetylglucosaminidase. The data is presented as mean ± SD for *n* = 6. ^###^(*p* < 0.001) indicating significant difference compared to control group, while ^*∗*^(*P* < 0.05), ^*∗∗*^(*P* < 0.01), and ^*∗∗∗*^(*p* < 0.001) indicate significant difference compared to arthritic group.

FCA resulted in increased acid phosphatase levels in arthritic group as compared to control group (0.8450 ± 0.0432 vs. 0.2233 ± 0.0463). Both high dose of *A. occidentale* and piroxicam showed a significant reduction in acid phosphatase levels as compared to arthritic group (0.4300 ± 0.0540 vs. 0.845 ± 0.0432) and (0.3583 ± 0.062 vs. 0.845 ± 0.043), respectively. Low dose of *A. occidentale* did not show any significant reduction in acid phosphatase levels compared to arthritic group (0.7667 ± 0.043 vs. 0.845 ± 0.0432). There was no significant difference between the effects of high-dose *A. occidentale* and piroxicam (0.430 ± 0.054 vs. 0.3583 ± 0.062) (Figure 4(a)).

#### 3.10.2. *A. occidentale* Significantly Suppressed *β*-Galactosidase Levels

Elevated levels of *β*-galactosidase were found in arthritic group as compared to control group (3.500 ± 0.1814 vs. 1.867 ± 0.0778). Both high dose of *A. occidentale* and piroxicam showed a significant reduction in *β*-galactosidase levels as compared to arthritic group (2.542 ± 0.1532 vs. 3.500 ± 0.1814) and (2.372 ± 0.1038 vs. 3.500 ± 0.1814), respectively. Low dose of *A. occidentale* did not show any significant reduction in *β*-galactosidase levels compared to arthritic group (3.358 ± 0.1932 vs. 3.500 ± 0.1814). There was no significant difference between the effect of high-dose *A. occidentale* and piroxicam (2.542 ± 0.1532 vs. 2.372 ± 0.1038) (Figure 4(b)).

#### 3.10.3. *A. occidentale* Significantly Attenuated *β*-Glucuronidase Levels

Results indicated high levels of *β*-glucuronidase in arthritic group as compared to control group (5.737 ± 0.2317 vs. 2.460 ± 0.2536). Both high dose of *A. occidentale* and piroxicam treatments resulted in a significant reduction in *β*-glucuronidase levels as compared to arthritic group (2.918 ± 0.1569 vs. 5.737 ± 0.2317) and (3.163 ± 0.3574 vs. 5.737 ± 0.2317), respectively. Low dose of *A. occidentale* showed no significant reduction in *β*-glucuronidase levels comparing to arthritic group (5.327 ± 0.4614 vs. 5.737 ± 0.2317). There was no significant difference between the effect of high-dose *A. occidentale* and piroxicam (2.918 ± 0.1569 vs. 3.163 ± 0.3574) (Figure 4(c)).

#### 3.10.4. *A. occidentale* Significantly Alleviated *N*-Acetyl Glucosaminidase Levels

Arthritic group showed increased levels of *N*-acetyl glucosaminidase as compared to control group (2.957 ± 0.0948 vs. 1.218 ± 0.0741). Both low dose and high dose of *A. occidentale* treatments resulted in a significant reduction in *N*-acetyl glucosaminidase levels as compared to arthritic group (2.707 ± 0.2229 vs. 2.957 ± 0.0948) and (2.047 ± 0.1211 vs. 2.957 ± 0.0948), respectively. Piroxicam also significant reduced in *N*-acetyl glucosaminidase levels compared to arthritic group (2.257 ± 0.1618 vs. 2.957 ± 0.0948). There was no significant difference between the effect of high-dose *A. occidentale* and piroxicam (2.047 ± 0.1211 vs. 2.257 ± 0.1618) (Figure 4(d)).

#### 3.10.5. *A. occidentale* Significantly Decreased Collagenase Levels

**Figure 5:**
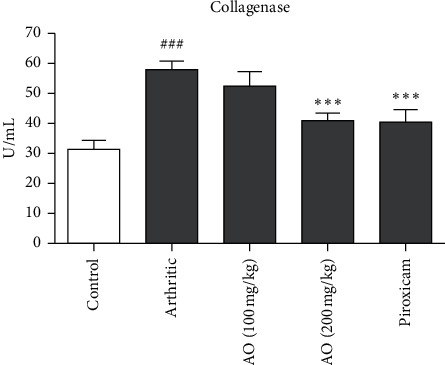
Treatment with *A. occidentale* leaves extract significantly reduced collagenase. The data is presented as mean ± SD for *n* = 6. ^###^(*p* < 0.001) indicating significant difference compared to control group, while ^*∗∗∗*^(*p* < 0.001) indicates significant difference compared to arthritic group.

Results showed increased levels of collagenase in arthritic group as compared to control group (58.32 ± 3.308 vs. 31.25 ± 3.204). Both high dose of *A. occidentale* and piroxicam treatments resulted in a significant reduction in collagenase levels as compared to arthritic group (40.60 ± 2.109 vs. 58.32 ± 3.308) and (41.21 ± 3.967 vs. 58.32 ± 3.308), respectively. Low dose of *A. occidentale* showed no significant reduction in collagenase levels compared to arthritic group (52.28 ± 5.455 vs. 58.32 ± 3.308). There was no significant difference between the effect of high-dose *A. occidentale* and piroxicam (40.60 ± 2.109 vs. 41.21 ± 3.967) (Figure 5).

## 4. Discussion

Cashew leaves are known for their medicinal properties. The plant is used by the traditional practitioners as anti-inflammatory agent [29]. Various studies using different models of inflammation have validated the anti-inflammatory properties of cashew [14, 15]. Current study assessed the antiarthritic effect of cashew leaves extract using FCA-induced arthritic rat model. Adjuvant-induced arthritic model is a well-established model and has been used in several studies to determine the potential therapeutic targets and pathogenesis of RA. The role of inflammatory mediators and serological and pathological changes in adjuvant-induced arthritic model is considered similar to human rheumatoid arthritis [30, 31]. Synovial joints are particularly affected in rheumatoid arthritis. The development of synovial cell hyperplasia, inflammation, bone erosion, and pannus formation are considered as characteristic histopathological features of rheumatoid arthritis [3, 32]. Chronic inflammation of synovial tissues is the major reason for the damage inflicted to bone and cartilage [17]. Current study demonstrated that the treatment with higher dose of *A. occidentale* significantly attenuated arthritic development, bone erosion, and pannus formation which might be ascribed to anti-inflammatory effects of plant extracts. Reference drug used was piroxicam because it is usually prescribed in the treatment of arthritis and is known to ameliorate adjuvant-induced arthritis through its immunomodulatory and anti-inflammatory activities [33, 34].

CRP is known as an acute phase protein and serves as an essential biomarker for different inflammatory, neoplastic, and degenerative disorders. Increased levels of CRP in blood are associated with almost all types of inflammatory disorders, especially in patients suffering from rheumatoid arthritis [35, 36]. RF is another important biomarker associated with various autoimmune and nonautoimmune diseases, especially rheumatoid arthritis. It is an antibody directed against the antigenic determinants on the Fc region of the immunoglobulin *G* [37]. RF has long been linked with the pathogenesis of RA and its induction is triggered by the formation of immune complexes during infection [38]. The elevated levels of CRP and RF in arthritic group in present study were found significantly reduced after treatment with both plant extract and piroxicam.

We found increase in TLC and platelet count in arthritic rats as compared with control group. This may be explained as the result of induction of immune response against attacking pathogen. Previous studies using adjuvant-induced arthritic model showed reduction in RBC count and Hb content in arthritic rats. This decline in hematological markers is an indicative of anemic condition. The anemia may be attributed to different reasons, e.g., failure of iron storage in reticuloendothelial system and synovial tissues and lack of adequate cell production due to failure of bone marrow functioning [18, 39, 40]. Treatment with *A. occidentale* normalized all the hematological parameters and no significant difference was found between the effects of plant extract and piroxicam.

We also evaluated the potential effects of *A. occidentale* extract on the levels of different lysosomal enzymes. Lysosomal enzymes have particular importance in rheumatoid arthritis. Lining cells of the synovial membrane and synovial fluid leucocytes were presumed to be the source of the lysosomal enzymes [41]. Acid phosphatase is one of the lysosomal enzymes and is considered responsible for the destruction of cartilage in arthritic condition. Levels of acid phosphatase are increased due to reduced stability of lysosomes in adjuvant-induced arthritis. The enhanced activity of acid phosphatase is an indicative of persistent inflammation [42, 43]. *β*-Galactosidase and *β*-glucuronidase are other lysosomal enzymes which cleave glycosidic bonds in proteoglycans and glycoproteins resulting in destruction of articular cartilage. A previous study showed that the activities of these exoglycosidase enzymes in both synovial fluid and serum of the arthritic patients were significantly increased [44]. *N*-acetylglucosaminidase is also a marker of inflammation and causes the hydrolysis of *N*-acetylglucosamine residues from *N*-acetylglucosaminides [45]. It normally exists in plasma and its levels are found increased in various inflammatory disorders like arthropathies and chronic obstructive pulmonary diseases. In addition, enhanced levels are found in liver disease, diabetes mellitus, and cancer [46]. These results are in accordance with the outcome of this study which also showed enhanced levels of *N*-acetylglucosaminidase, *β*-glucuronidase, acid phosphatase, and *β*-galactosidase, and in rat serum after induction of arthritis. Treatment with plant extract significantly attenuated the levels of these lysosomal enzymes as compared with arthritic group.

Irreversible joint destruction is a characteristic of RA. Interstitial collagens are present in high content in joints and are critical structural targets of different enzymes. Although the presence of type-I collagen dominates in cartilaginous matrix, type-II collagen is largely found in extracellular matrix of bone, tendons, and ligaments. Collagenase acts by cleaving collagen within triple helical domains, thus playing an important role in RA-associated tissue destruction [47]. We found that treatment with *A. occidentale* extract significantly reduced collagenase levels as compared with arthritic group. The data showed that the amelioration of FCA-induced arthritis as evidenced by reduction in arthritic score and histopathological score might be attributed to the suppression of various tissue destructive enzyme levels.

A lot of studies have been conducted to evaluate the qualitative and quantitative phytochemical constituents of *A. occidentale* and are summarized in the form of a comprehensive review by [48]. Different vitamins and functional biofactors were reported in the cashew leaves, e.g., vitamin C, vitamins B2 and B3, thiamine, methyl gallute, leucocyanidin, leucodelphinidin, etc. The presence of fatty acids, like *β*-sitosterol, stigmasterol, etc., in cashew leaves has also been documented. Various studies revealed the presence of a number of phenolic compounds in the cashew leaves, e.g., 2-hydroxy-6-pentadecylbenzoic acid, hyperoside, amentoflavone derivate, myricetin-O-glycoside, kaempferol-3-O-xyloside, quercetin-3-O-xyloside, quercetin-3-O-arabinofuranoside, quercetin-3-O-arabinopyranoside, quercetin-3-O-rutinoside, etc. [48]. In another study, fresh cashew leaves were subjected to steam distillation for the evaluation of essential oils and found *β*-phellandrene + limonene (17.5%), methyl chavicol (11.4%), germacrene B (8%), and trans-*α*-bergamotene (7.9%) in the dominant quantities [49]. Many of these constituents are known to possess anti-inflammatory and antioxidant properties, which might be responsible for the amelioration of rheumatoid arthritis in current study.

## 5. Conclusion

The study suggests that ethanolic extract of leaves of *Anacardium occidentale* possesses significant antiarthritic properties in rats with FCA-induced arthritis and is attributed to the inhibition of lysosomal enzymes and collagenase. Notably, higher dose of *Anacardium occidentale* (200 mg/kg b.w.) exhibited better anti-inflammatory effects as compared to low dose (100 mg/kg b.w.).

## Figures and Tables

**Table 1:** Treatment with *A. occidentale* nearly normalized all the hematological parameters.

Parameters	Groups				
	Control	Arthritic	AO (100 mg/kg)	AO (200 mg/kg)	Piroxicam
TLC (10^3^)	7.305 ± 0.388	9.293 ± 0.569^###^	8.017 ± 0.321^*∗∗*^	8.017 ± 0.321^*∗∗∗*^	7.807 ± 0.663^*∗∗∗*^
Neutrophil (%)	52.66 ± 3.369	66.78 ± 2.660^###^	60.43 ± 3.972^*∗*^	57.94 ± 3.052^*∗∗∗*^	54.50 ± 3.355^*∗∗∗*^
Lymphocytes (%)	31.18 ± 0.608	33.66 ± 0.708^###^	33.27 ± 1.693	32.02 ± 0.551^*∗*^	31.96 ± 0.532^*∗*^
Monocytes (%)	1.610 ± 0.133	2.412 ± 0.200^###^	2.303 ± 0.1720	2.088 ± 0.117^*∗*^	2.032 ± 0.146^*∗∗*^
Basophils (%)	0.8050 ± 0.034	0.8500 ± 0.052	0.8400 ± 0.030	0.8267 ± 0.043	0.8117 ± 0.0256
Platelet count (10^3^)	4.350 ± 0.250	5.567 ± 0.325^###^	5.133 ± 0.189	4.908 ± 0.364^*∗∗*^	4.882 ± 0.184^*∗∗*^
RBC (10^6^)	5.827 ± 0.325	4.163 ± 0.405^###^	4.450 ± 0.424	5.057 ± 0.364^*∗∗*^	4.963 ± 0.144^*∗∗*^
Hb (g/dL)	14.66 ± 0.3985	11.67 ± 0.8179^###^	12.79 ± 0.3164^*∗*^	13.36 ± 0.4298^*∗∗∗*^	13.02 ± 0.6174^*∗*^

^###^ *P* < 0.001 indicating significant difference compared to control group, ^*∗*^*P* < 0.05, ^*∗∗*^*P* < 0.01, and ^*∗∗∗*^*P* < 0.001 indicating significant difference compared to arthritic group.

## Data Availability

Data are available from the corresponding author upon request.

## Abbreviations

CRP: C-reactive protein
TLC: Total leukocyte count
DLC: Differential leukocyte count
Rf: Rheumatoid factor
Hb: Hemoglobin
RBC: Red blood cells
FCA: Freund's complete adjuvant.
